# Prescribing Prevalence of Medications With Potential Genotype-Guided Dosing in Pediatric Patients

**DOI:** 10.1001/jamanetworkopen.2020.29411

**Published:** 2020-12-14

**Authors:** Laura B. Ramsey, Henry H. Ong, Jonathan S. Schildcrout, Yaping Shi, Leigh Anne Tang, J. Kevin Hicks, Nihal El Rouby, Larisa H. Cavallari, Sony Tuteja, Christina L. Aquilante, Amber L. Beitelshees, Daniel L. Lemkin, Kathryn V. Blake, Helen Williams, James J. Cimino, Brittney H. Davis, Nita A. Limdi, Philip E. Empey, Christopher M. Horvat, David P. Kao, Gloria P. Lipori, Marc B. Rosenman, Todd C. Skaar, Evgenia Teal, Almut G. Winterstein, Aniwaa Owusu Obeng, Daria Salyakina, Apeksha Gupta, Joshua Gruber, Jennifer McCafferty-Fernandez, Jeffrey R. Bishop, Zach Rivers, Ashley Benner, Bani Tamraz, Janel Long-Boyle, Josh F. Peterson, Sara L. Van Driest

**Affiliations:** 1 Department of Pediatrics, College of Medicine, University of Cincinnati, Cincinnati, Ohio; 2 Divisions of Research in Patient Services and Clinical Pharmacology, Cincinnati Children’s Hospital Medical Center, Cincinnati, Ohio; 3 Vanderbilt Institute for Clinical and Translational Research, Vanderbilt University Medical Center, Nashville, Tennessee; 4 Department of Biostatistics, Vanderbilt University Medical Center, Nashville, Tennessee; 5 Department of Biomedical Informatics, Vanderbilt University Medical Center, Nashville, Tennessee; 6 Department of Individualized Cancer Management, H. Lee Moffitt Cancer Center and Research Institute, Tampa, Florida; 7 Department of Pharmacotherapy and Translational Research, University of Florida, Gainesville; 8 James Winkle College of Pharmacy, University of Cincinnati, Cincinnati, Ohio; 9 Department of Medicine, Perelman School of Medicine, University of Pennsylvania, Philadelphia; 10 Skaggs School of Pharmacy and Pharmaceutical Sciences, University of Colorado, Aurora; 11 Department of Medicine, University of Maryland, Baltimore; 12 Department of Emergency Medicine, University of Maryland, Baltimore; 13 Center for Pharmacogenomics and Translational Research, Nemours Children’s Health System, Jacksonville, Florida; 14 Nemours Research Institute, Nemours Children’s Health System, Jacksonville, Florida; 15 Informatics Institute, University of Alabama at Birmingham; 16 Department of Neurology, University of Alabama at Birmingham; 17 Department of Pharmacy and Therapeutics, School of Pharmacy, University of Pittsburgh, Pittsburgh, Pennsylvania; 18 Department of Critical Care Medicine, School of Medicine, University of Pittsburgh, Pittsburgh, Pennsylvania; 19 Department of Medicine, School of Medicine, University of Colorado, Aurora; 20 University of Florida Health and University of Florida Health Sciences Center, Gainesville; 21 Department of Pediatrics, Indiana University School of Medicine, Indianapolis; 22 Department of Pediatrics, Ann & Robert H. Lurie Children’s Hospital of Chicago, Chicago, Illinois; 23 Department of Medicine, Indiana University School of Medicine, Indianapolis; 24 Regenstrief Institute, Indianapolis, Indiana; 25 Department of Pharmaceutical Outcomes and Policy and Center for Drug Evaluation and Safety, University of Florida, Gainesville; 26 The Charles Bronfman Institute for Personalized Medicine, Departments of Medicine and Genetics and Genomic Sciences, Icahn School of Medicine at Mount Sinai, New York, New York; 27 Personalized Medicine Initiative, Nicklaus Children’s Health System, Miami, Florida; 28 Department of Experimental and Clinical Pharmacology, University of Minnesota College of Pharmacy, Minneapolis; 29 Department of Psychiatry, University of Minnesota Medical School, Minneapolis; 30 Department of Pharmaceutical Care and Health Systems, University of Minnesota College of Pharmacy, Minneapolis; 31 Clinical and Translational Science Institute, University of Minnesota, Minneapolis; 32 School of Pharmacy, University of California, San Francisco; 33 Department of Medicine, Vanderbilt University Medical Center, Nashville, Tennessee; 34 Department of Pediatrics, Vanderbilt University Medical Center, Nashville, Tennessee

## Abstract

**Importance:**

Genotype-guided prescribing in pediatrics could prevent adverse drug reactions and improve therapeutic response. Clinical pharmacogenetic implementation guidelines are available for many medications commonly prescribed to children. Frequencies of medication prescription and actionable genotypes (genotypes where a prescribing change may be indicated) inform the potential value of pharmacogenetic implementation.

**Objective:**

To assess potential opportunities for genotype-guided prescribing in pediatric populations among multiple health systems by examining the prevalence of prescriptions for each drug with the highest level of evidence (Clinical Pharmacogenetics Implementation Consortium level A) and estimating the prevalence of potential actionable prescribing decisions.

**Design, Setting, and Participants:**

This serial cross-sectional study of prescribing prevalences in 16 health systems included electronic health records data from pediatric inpatient and outpatient encounters from January 1, 2011, to December 31, 2017. The health systems included academic medical centers with free-standing children’s hospitals and community hospitals that were part of an adult health care system. Participants included approximately 2.9 million patients younger than 21 years observed per year. Data were analyzed from June 5, 2018, to April 14, 2020.

**Exposures:**

Prescription of 38 level A medications based on electronic health records.

**Main Outcomes and Measures:**

Annual prevalence of level A medication prescribing and estimated actionable exposures, calculated by combining estimated site-year prevalences across sites with each site weighted equally.

**Results:**

Data from approximately 2.9 million pediatric patients (median age, 8 [interquartile range, 2-16] years; 50.7% female, 62.3% White) were analyzed for a typical calendar year. The annual prescribing prevalence of at least 1 level A drug ranged from 7987 to 10 629 per 100 000 patients with increasing trends from 2011 to 2014. The most prescribed level A drug was the antiemetic ondansetron (annual prevalence of exposure, 8107 [95% CI, 8077-8137] per 100 000 children). Among commonly prescribed opioids, annual prevalence per 100 000 patients was 295 (95% CI, 273-317) for tramadol, 571 (95% CI, 557-586) for codeine, and 2116 (95% CI, 2097-2135) for oxycodone. The antidepressants citalopram, escitalopram, and amitriptyline were also commonly prescribed (annual prevalence, approximately 250 per 100 000 patients for each). Estimated prevalences of actionable exposures were highest for oxycodone and ondansetron (>300 per 100 000 patients annually). CYP2D6 and CYP2C19 substrates were more frequently prescribed than medications influenced by other genes.

**Conclusions and Relevance:**

These findings suggest that opportunities for pharmacogenetic implementation among pediatric patients in the US are abundant. As expected, the greatest opportunity exists with implementing *CYP2D6* and *CYP2C19* pharmacogenetic guidance for commonly prescribed antiemetics, analgesics, and antidepressants.

## Key Points

**Question:**

What is the opportunity for genotype-guided prescribing among pediatric patients in the US?

**Findings:**

In this serial cross-sectional study of annual prescribing data at 16 health systems representing approximately 2.9 million pediatric patients per year from 2011 to 2017, the annual prevalence of exposure to at least 1 Clinical Pharmacogenetics Implementation Consortium level A drug ranged from 7987 to 10 629 per 100 000 pediatric patients, with increasing prevalence before reaching a plateau in 2014. The medications with the highest potential for actionability were analgesics (oxycodone, codeine, and tramadol), the antiemetic ondansetron, and antidepressants (citalopram, escitalopram, and amitriptyline).

**Meaning:**

These findings suggest that ample opportunity exists for genotype-guided prescribing among pediatric patients in the US, especially for drugs metabolized by CYP2D6 or CYP2C19.

## Teaser

This cross-sectional study assesses potential opportunities for genotype-guided prescribing in pediatric populations among multiple health systems by examining the prevalence of prescriptions for each drug with the highest level of evidence and estimating the prevalence of potentially actionable prescribing decisions.

## Introduction

Pharmacogenetics is a key component of precision medicine that uses genetic information to guide drug selection and dosing decisions.^1^ More than 100 commercially available drugs in the US contain pharmacogenetic information in the US Food and Drug Administration (FDA) label, including therapeutic management recommendations and warnings about the potential effects on drug safety, response, or potential alterations in pharmacokinetic parameters.^2,3^ The Clinical Pharmacogenetics Implementation Consortium (CPIC) publishes evidence-based, drug-centric, peer-reviewed guidelines for how to translate genetic test results into actionable prescribing decisions.^4,5,6^ During the last decade, CPIC has published guidelines on more than 35 gene-drug pairs as CPIC level A, indicating that a prescribing action is recommended when genotype information is available and that the preponderance of evidence is high or moderate in favor of changing prescribing.^7^

Although most pharmacogenetic research to date has focused on adults, the potential benefits of genotype-guided therapy in children are increasingly recognized.^8,9,10^ Many CPIC guidelines suggest application of genotype-guided therapy in both adults and children, whereas some CPIC guidelines contain unique recommendations for children (eg, voriconazole, atomoxetine, and warfarin), owing to ontogeny or other pediatric considerations.^11,12,13^ There is at least 1 example of a greater effect of a pharmacogenetic variant in children than adults that involves the drug transporter SLCO1B1 and simvastatin.^14^ Thus, the opportunity exists for more personalized pharmacotherapy in the pediatric population; however, widespread clinical implementation is challenging.

Data on trends of the prevalence of relevant prescriptions over time are crucial to informing pharmacogenetic implementation. The utility of pharmacogenetic testing depends on the frequency of prescribed medications with an actionable association and can rapidly change with the introduction of new drugs, the availability of generics or different formulations, changes in pricing, revised FDA guidance, and decisions by health care payers, health care institutions, and prescribers. Prescribing trends can also inform which genes to test, the opportunity for reuse of results during a lifetime (when a second drug associated with the same gene is prescribed), and the use of multigene panel-based tests.

In 2013, the National Institutes of Health funded the IGNITE (Implementing Genomics in Practice) Network, including 6 member sites, 15 affiliate sites, and a coordinating center, to support the development, investigation, and dissemination of genomic-guided practice models that seamlessly integrate such data into the electronic health record (EHR).^15^ We leveraged the IGNITE Network to conduct a retrospective, longitudinal analysis of the annual prevalence of prescriptions for CPIC level A drugs among pediatric patients (aged <21 years) across multiple types of health systems and population demographics. Our objective was to assess potential opportunities for genotype-guided prescribing in pediatric populations among multiple health systems by examining the prevalence of prescriptions for each CPIC level A drug and estimating the prevalence of potentially actionable prescribing decisions.

## Methods

### Overview

This serial, cross-sectional study assessed prescribing prevalence patterns across 16 health systems. In lieu of transferring patient data from each site, each site summarized individual-level data in a standardized format using demographic, temporal, drug, and gene characteristics. Aggregate data were then provided for central analysis. We estimated prescribing prevalences for each CPIC level A drug per site and across calendar years. Because some sites did not have data for all years, we used logistic regression to estimate prevalences over time for each site. We then summarized results to calculate across-site prevalence of exposure for each drug, drug class, and gene of interest. Additional details on the methods are available in the eMethods in the Supplement. This report adheres to the Strengthening the Reporting of Observational Studies in Epidemiology (STROBE) guidelines for cross-sectional studies.^16^

### Participating Sites

The work reported was developed within the IGNITE Pharmacogenetics Working Group.^17^ All member and affiliate sites with pediatric data were invited to participate in the study. All participating sites signed a collaborative agreement and obtained approval from their respective institutional review boards for all data abstraction and reporting. The need for informed consent was waived for this use of deidentified data.

### Study Medications and Genes

At the time the study analysis was begun (June 5, 2018), the list of 39 CPIC level A drugs and 20 associated genes (eTable 1 in the Supplement) was obtained from the CPIC website (https://cpicpgx.org/genes-drugs/). Because 1 level A drug, tropisetron, is not available in the US, we studied 38 medications. Three medications (warfarin, clopidogrel, and simvastatin) had clinical pharmacogenetic testing available during the study time frame at some sites; thus, data about alternative therapies were also obtained to enable accurate assessment of pharmacogenetic opportunity. For each drug, the RxNorm term for the ingredient was used to generate a list of all medications and formulations, including combination therapies, containing that ingredient. Formulations without systemic exposure (eg, topical formulations) were excluded. The list of drug ingredients, RxNorm terms, and all compiled generic and brand-name medications containing CPIC level A drugs from an Epic Clarity system (EPIC Systems Corporation) was provided to each site for subsequent site-level validation and adjustment as needed to support complete data capture. Prescription data collected included both inpatient and outpatient exposures at all sites except as indicated in eTable 2 in the Supplement.

### Data Collection and Aggregation

Collection of data including demographics and prescribing and encounter information was completed at each site and uniformly structured according to the study data dictionary. The annual number of unique individuals with encounters where a prescription may have been provided was reported by each site to serve as the denominator for prevalence estimates. R scripts were developed, tested, and disseminated to aggregate individual-level prescription data by specific characteristics. Similarly, corresponding R scripts were developed to capture the number of unique patients with at least 1 encounter (within each subgroup as defined by patient characteristics) to estimate annual prescribing prevalence; each site submitted aggregate data for analyses.

### Statistical Analysis

Data were analyzed from June 5, 2018, to April 14, 2020. For demographic characteristics at each site, we calculated summary statistics on an annual basis and summarized prevalence estimates as the median across calendar years. We then combined site-specific summaries to obtain overall summaries as shown in Table 1.

**Table 1.** Characteristics of the Patient Populations Across 16 Sites Observed From 2011 to 2017^a^

Characteristic	Data
No. of sites	16
No. of academic medical centers	12
No. of community hospitals or clinic systems	4
Age, y	
25th Percentile	3.00 (2.00-3.75)
50th Percentile	8.00 (7.00-10.00)
75th Percentile	14.00 (12.00-16.25)
Female, %	50.7 (47.4-67.7)
Race/ethnicity, %	
White	62.3 (12.2-86.9)
Black	18.0 (6.8-70.2)
Asian	1.4 (0.2-11.3)
American Indian or Alaska Native	0.2 (0.0-1.0)
Pacific Islander	0.1 (0.0-1.1)
Other or unknown	11.1 (2.6-58.2)
Unique patients with encounters per year	96 597 (4790-799 964)
Sum of medians across sites	2 866 887
Unique patients with target prescriptions per year^b^	6057 (238-38 230)
Sum of medians across sites	197 409

^a^ Unless otherwise indicated, data are expressed as median (range). Summary statistics were derived from site-level, across-year medians. For example, the median (range) of unique patients with encounters was derived by calculating the site-specific median number of encounters per year across observed years and then calculating the median (range) of the site-specific median values. For the 25th percentile of age summary, at each site, we calculated the 25th percentile of age each year and then used the median of those values. The median (range) is reported in the table as the across-sites median (range) of the site-specific median values for the 25th percentiles.

^b^ Target prescriptions defined as Clinical Pharmacogenetics Implementation Consortium level A drugs or alternative medications within the class.

#### Prescribing Patterns Over Time

Not every site was able to provide data for all years included in the analysis. Thus, we used logistic regression to estimate site-specific prevalence of prescriptions from 2011 to 2017 for each of the following: (1) any CPIC level A medication; (2) at least 1, 2, 3, and 4 CPIC level A medications; (3) distinct classes of CPIC level A medications (eg, analgesics, statins, anticoagulants); (4) individual CPIC level A medications; and (5) medications with associated genes. Each model included site-by-year interactions, allowing us to estimate the annual site-specific prescription prevalences for each medication, which we combined across sites to obtain overall, annual prescription prevalences, as described below. Missing data for 2 or fewer consecutive years were assumed to be missing at random. When data were missing for more than 2 consecutive years, the time trend for the site was removed to avoid excessive extrapolation with a nonlinear function. Annual prescription prevalence is expressed per 100 000 unique individuals with at least 1 inpatient or outpatient encounter that year. Primary data analyses combined site-year prevalences across sites with each site weighted equally. Because of site-to-site variability in sample sizes (see eTable 2 in the Supplement), we performed a sensitivity analysis using patient weighting, which weighted sites in proportion to the number of patients with encounters at that site.

One challenge with these analyses was that not all sites contributed prescribing data for all years. Because the availability, or lack thereof, of prescribing data relied on an operational and compatible EHR system and was unlikely to be related to prescribing patterns themselves, we assumed the data were missing at random when sites were missing data for 1 or 2 consecutive years. If more than 2 consecutive years were missing within a site, we removed the site-specific time trend and estimated a single site-specific prevalence. Similarly, if there were fewer than 20 prescriptions for a medication in any year within a site, we removed the time trend for that site to avoid highly variable estimates and instead estimated a single site-specific prevalence across all years.

#### Prescribing Patterns by Demographic Characteristics

To examine the association of sex, race, and age with prescribing patterns, we used methods similar to those described above and detailed in the eMethods in the Supplement. For example, to estimate prescribing patterns across the age distribution, we removed terms for year and the site-by-year interaction and added a term for age using restricted cubic spline functions to the models above to permit nonlinear age trends. To estimate the frequency of actionable exposures based on demographic data, the frequency of actionable phenotypes by ancestry was extracted from the gene’s supplemental table on the CPIC website.^7^ For CYP2D6, activity scores of 1 were assigned the normal metabolizer phenotype, because this work predated the updated *CYP2D6* genotype-to-phenotype translation guideline.^18^

## Results

### Study Cohort

Data from approximately 2.9 million pediatric patients (median age, 8 [interquartile range, 2-16] years; 50.7% female and 49.3% male; 62.3% White) were analyzed for a typical calendar year. Table 1 and eTable 2 in the Supplement describe characteristics of the 16 participating sites and demographic data for the pediatric populations with encounters. Twelve of the sites are academic medical centers, 2 of which contributed data from a community hospital or clinic system. Four sites are community hospitals or clinic systems. Six of the sites provided data from a free-standing children’s hospital. The 16 sites ranged in pediatric patient volume (median number of unique patients with encounters per year) from 4790 to 799 964 per year; the sum of these medians across all 16 sites is 2 866 887 individuals, representing the estimated annual number of individuals observed. The median number of unique patients per year with prescriptions for CPIC level A drugs or their alternatives ranged from 238 to 38 230; the sum of these annual medians is 197 409 individuals.

### Prevalence of Exposure to CPIC Level A drugs

**Figure 1.**
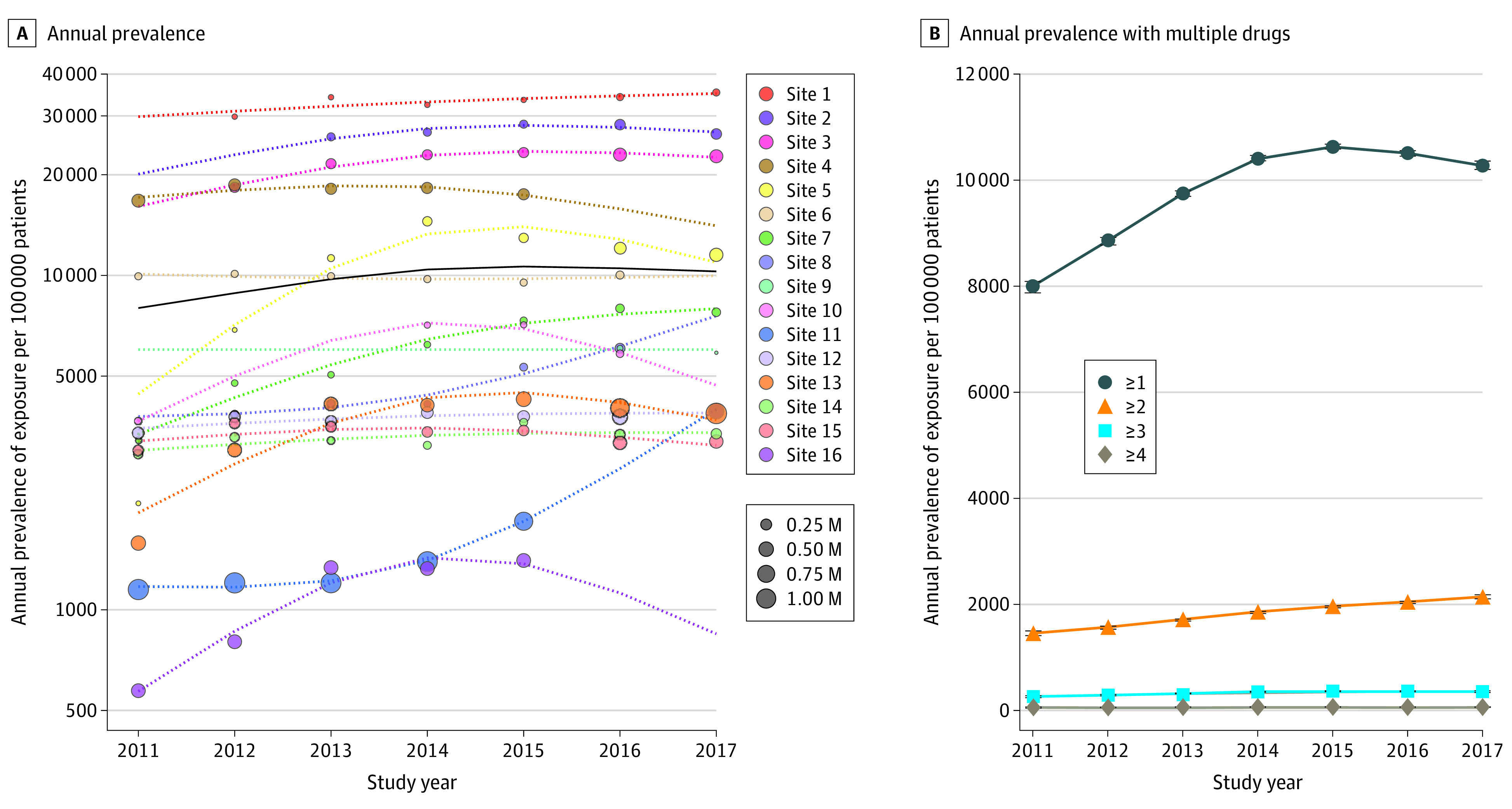
Annual Prevalence of Exposure to at Least 1 Clinical Pharmacogenetics Implementation Consortium (CPIC) Level A Medication by Site and to 1 or More CPIC Level A Medications A, Each circle represents the observed prevalence of exposure for a given site on a log scale. Circles are absent for years when data were not available. The size of the circle is proportional to the number of patients who experienced at least 1 encounter in that year. The dotted lines represent the prevalence of exposure estimated from the model fit. The mean prevalence of exposure across all sites is shown by the solid black line. The 95% CIs for the mean is filled in gray but may be too narrow to observe. B, On a linear scale, the mean annual prevalence of exposure is stratified by the number of CPIC level A medications prescribed. The prevalence of exposure was estimated from the model. The whiskers indicate 95% CIs. M indicates million.

Figure 1A depicts the estimated annual prevalence of exposure to at least 1 CPIC level A drug per 100 000 patients, which ranged from 7987 to 10 629 during the study years. This prevalence increased from 7987 in 2011 to 10 415 in 2014, where it remained stable through 2017 (±200) (Figure 1A). The most prescribed CPIC level A drug was the antiemetic ondansetron (annual prevalence of exposure, 8107 [95% CI, 8077-8137] per 100 000 patients [Table 2]). The opioids tramadol (prescription prevalence, 295 [95% CI, 273-317] per 100 000 patients annually), codeine (571 [95% CI, 557-586] per 100 000 patients annually), and oxycodone (2116 [95% CI, 2097-2135] per 100 000 patients annually) were commonly prescribed. The antidepressants citalopram, escitalopram, and amitriptyline were also commonly prescribed (annual prevalence, approximately 250 per 100 000 patients each). There was wide variation across sites, from persistently less than 5000 to 30 000 or more prescriptions per 100 000 patients. In the sensitivity analysis using by-patient weighting, the estimated annual prevalences across all sites were lower (larger sites had lower prescription prevalence [eg, site 11]) and ranged from 5275 to 6892. When exposure to multiple CPIC level A drugs was assessed, we observed that the annual prevalence of exposure to at least 2 CPIC level A drugs increased from 1468 (in 2011) to 2157 (in 2017) per 100 000 patients (Figure 1B). The age of exposure to at least 1 CPIC level A drug, assessed using 2015 data, indicated an early peak around 5 years of age and a second increase in exposure around 15 years of age (eFigure 1 in the Supplement). Examination of specific drugs revealed that ondansetron exposure peaked at 4 to 5 years of age, and escitalopram exposure peaked at approximately 15 years of age (eFigure 2 in the Supplement).

**Table 2.** Annual Estimated Prevalences per 100 000 Patients of Actionable Exposures

Medication by class	Annual prescription prevalence per 100 000 patients (95% CI)	Gene	Actionable phenotype^a^	Annual actionable gene-drug interaction prevalence per 100 000 patients (95% CI)
Antiemetic				
Ondansetron	8107 (8077-8137)	*CYP2D6*	UM	325 (324-327)
Analgesic				
Oxycodone	2116 (2097-2135)	*CYP2D6*	PM, IM, UM	356 (352-359)
Codeine	571 (557-586)	*CYP2D6*	PM, IM, UM	98 (95-100)
Tramadol	295 (273-317)	*CYP2D6*	PM, IM, UM	53 (49-57)
Antidepressant				
Citalopram	283 (278-287)	*CYP2C19*	PM, RM, UM	94 (92-95)
Amitriptyline	272 (267-277)	*CYP2C19*	PM, RM, UM	90 (89-92)
Amitriptyline	272 (267-277)	*CYP2D6*	PM, IM, UM	46 (45-46)
Escitalopram	259 (255-264)	*CYP2C19*	PM, RM, UM	86 (84-87)

Abbreviations: IM, intermediate metabolizer; PM, poor metabolizer; RM, rapid metabolizer; UM, ultrarapid metabolizer.

^a^ CYP2D6 IM phenotype does not include the activity score of 1 or the updated activity score of the *10 allele as defined in the newest genotype-to-phenotype translation.^18^

### Prevalence of Exposure by Drug Class and Changes Over Time

**Figure 2.**
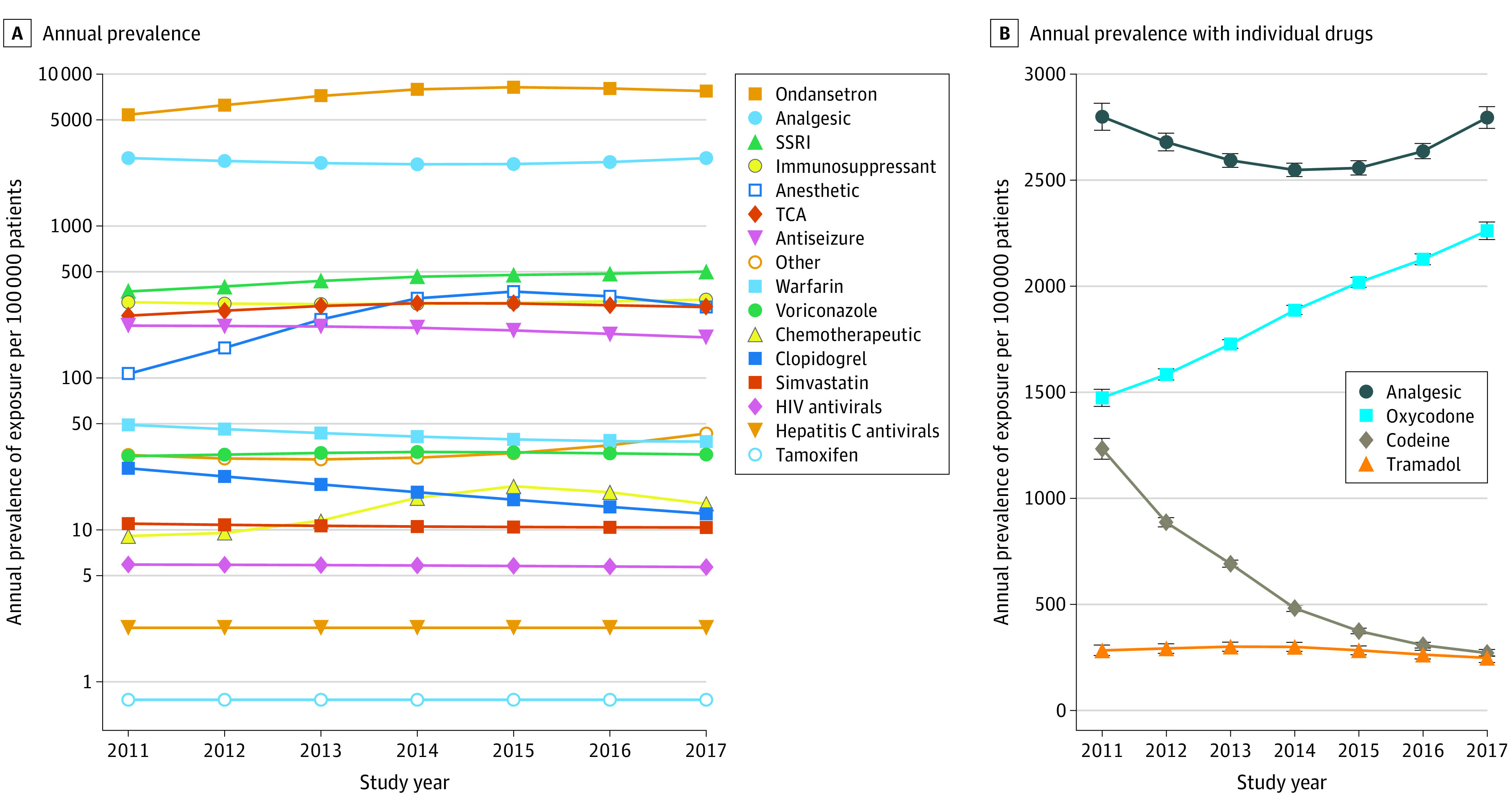
Annual Prevalence of Exposure to Clinical Pharmacogenetics Implementation Consortium (CPIC) Level A Medications, Stratified by Drug Class and Individual Analgesics A, The annual prevalence of exposure for each drug or drug class was estimated from the model and is plotted on a log axis. If a drug class only had a single included drug, that drug was listed instead of the drug class. For example, ondansetron is listed instead of antiemetic medications. B, Annual prevalence of exposure for analgesics is plotted on a linear scale. The estimated prevalence of exposure for all analgesics was taken from the drug class model in part A, whereas those for oxycodone, codeine, and tramadol were taken from the individual drug models. The whiskers indicate 95% CIs. Non-CPIC level A analgesics were not included. SSRI indicates selective serotonin reuptake inhibitor; TCA, tricyclic antidepressant.

Figure 2A illustrates the annual prevalence for each of the most commonly prescribed CPIC level A drug classes. From 2011 to 2017, ondansetron was prescribed at an annual prevalence ranging from 5399 to 8191 per 100 000 patients; opioid analgesics were prescribed at annual prevalences ranging from 2548 to 2799 per 100 000 patients. Medications used for more specific indications, such as antivirals, were prescribed for a much smaller proportion of patients. Most of the utilization trends remained constant over time, with notable exceptions being decreases in clopidogrel use (from 25 to 13 per 100 000 patients) and codeine use (from 1233 to 271 per 100 000 patients) and an increase in oxycodone use (from 1475 to 2262 per 100 000 patients) (Figure 2). When sites were stratified by primary population, pediatric health systems mirrored this trend for both opioids, whereas primarily adult health systems did not show significant changes in the type of opioid used across study years (eFigure 3 in the Supplement). Primarily pediatric health systems tended to have substantially higher prescribing prevalences for opioids (mean [SD], 3542 [163] per 100 000 patients) than did primarily adult health systems (mean [SD], 1721 [109] per 100 000 patients).

### Estimated Prevalence of Actionable Exposures

Based on the prevalence of exposure to each CPIC level A drug, the race/ethnicity data of the cohort, and known frequencies of actionable genotypes and phenotypes in the populations, we estimated the prevalence of medication exposure among individuals with the relevant actionable phenotype. In this cohort, a total of 1335 actionable exposures per 100 000 pediatric patients were eligible for a genotype-guided intervention, had information been available. Although ondansetron was the most frequently prescribed CPIC level A drug for children, it was not the drug with the highest prevalence of actionable exposure. The prevalence at which this drug was prescribed to patients with actionable phenotypes (actionable exposure) was 325 patients per 100 000 patients, because only the ultrarapid metabolizer phenotype is actionable. Oxycodone had the highest estimated prevalence of actionable prescribing, with 356 per 100 000 pediatric patients (Table 2). *CYP2D6* and *CYP2C19* accounted for 1170 of the 1335 actionable exposures per 100 000 patients (87.6%).

### Genes Associated With at Least 1 CPIC Level A Medication

**Figure 3.**
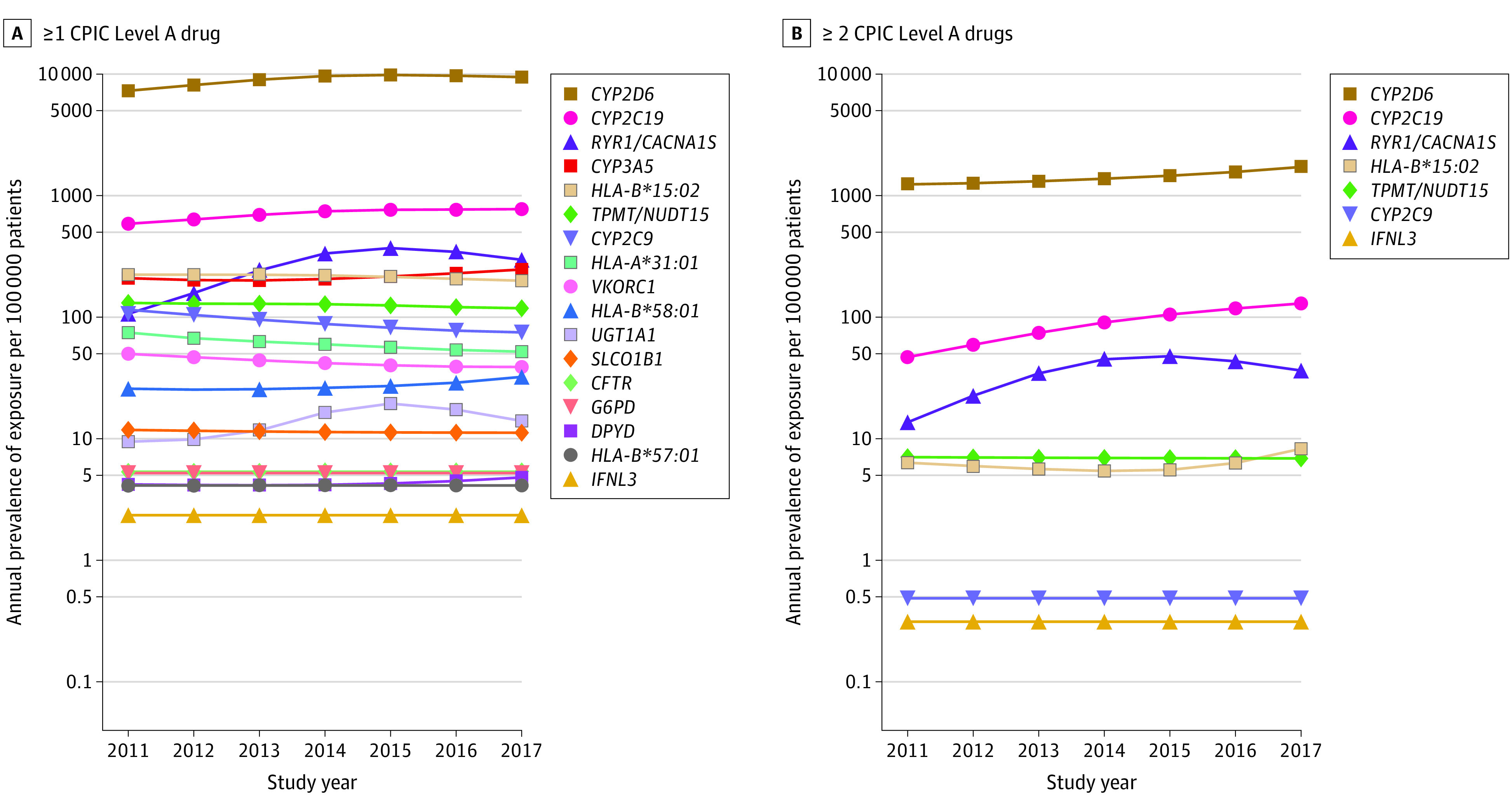
Annual Prevalence of Exposure to Clinical Pharmacogenetics Implementation Consortium (CPIC) Level A Medications Stratified by Gene A, Annual prevalence of exposure to at least 1 CPIC level A medication plotted on a log scale, stratified by the associated gene. B, Annual prevalence of exposure of at least 2 CPIC level A medications. The rate of exposure was estimated from the model and is displayed on a log scale.

Figure 3 shows the annual prevalence of exposure to CPIC level A medications stratified by the relevant gene associations. The most common gene associated with at least 1 prescribed CPIC level A medication prescription was *CYP2D6* (GeneBank 1565), with more than 5000 patients per 100 000 in all years studied (Figure 3A). *CYP2C19* (GeneBank 1557) and *RYR1* (GeneBank 6261)/*CACNA1S* (GeneBank 779) remained a distant second and third, respectively, with an order of magnitude fewer patients (≤775 and ≤370 patients per 100 000, respectively). These genes remained the most commonly associated when exposure to at least 2 CPIC level A medications was assessed (Figure 3B). The annual prevalence of exposure to multiple CPIC level A medications metabolized by CYP2D6 exceeded 1200 patients per 100 000 in all years studied.

## Discussion

To our knowledge, this is the first study to describe the prescribing of CPIC level A medications in pediatric patients among diverse sites in the US. The primary findings are that prescriptions for CPIC level A drugs are common (annual prescribing prevalence of approximately 8000 to 11 000 per 100 000 patients) in pediatrics and may be relevant to a broad spectrum of therapeutic areas, the importance of which may differ across institutions. Overall, we estimate that more than 1.3% of patients (>1300 per 100 000) would have potential recommendations for or may require drug selection or dosing changes based on current guidelines and pharmacogenetic testing results alone. Of the 20 relevant genes, *CYP2D6* and *CYP2C19* had the largest potential effect, because they affect commonly prescribed analgesics and antidepressants as well as the antiemetic ondansetron in the pediatric setting.

Our study focused on patients younger than 21 years, with most aged 3 to 14 years. Pediatric-specific analyses are important to quantify patterns of prescribing, highlight knowledge gaps, and quantify the potential effect of pharmacogenetics based on age, demographic characteristics, and drugs within pediatrics. One single-center study^19^ reported similar prevalences of prescribing, which differed from prevalences in adults.^20^ A 5-year study across Alberta, Canada,^21^ demonstrated that codeine-containing medications and ondansetron were the most commonly prescribed medications with pharmacogenetic-based prescribing guidelines in pediatric patients.

*CYP2D6-* and *CYP2C19-*associated drugs were the most commonly prescribed and represented most actionable exposures (87.6%). This includes all CPIC guideline recommendations, including those rated as optional in the guideline. Institutions have implemented clinical testing of these genes to guide drug or dose selection in pediatric patients.^22,23,24,25,26^ CPIC guidelines are available for the CYP2D6 substrates ondansetron,^27^ select opioids,^28,29^ tricyclic antidepressants,^30,31^ atomoxetine,^12^ tamoxifen,^32^ and selective serotonin reuptake inhibitors^33^; there are also CPIC guidelines for the CYP2C19 substrates tricyclic antidepressants,^30,31^ selective serotonin reuptake inhibitors,^33^ clopidogrel,^34,35^ and voriconazole.^11^ Initial data support the utility of *CYP2D6* or *CYP2C19* genotype-guided therapies for pediatrics^24,25,26^ and are anticipated from the IGNITE II Pragmatic Trials Network.^36^

*CYP2D6* and *CYP2C19* are also the genes most commonly associated with exposure to 2 or more CPIC level A drugs, indicating a potential for reuse of pharmacogenetic test results (particularly multigene tests), even within 1 year. Our observed prevalence of exposure to multiple CPIC level A drugs (<300 per 100 000 patients) is an underestimate of the true prevalence, because we were only able to aggregate data within single years. Opportunities for additional genotype-guided prescribing are likely to occur in subsequent years. For example, a child may have a pharmacogenetic test performed in response to prescription for a medication (eg, *CYP2C19* testing for escitalopram). If this test examines a panel of genes and these results are included in the EHR with associated clinical decision support, they could be used preemptively at the point of prescribing all future medications related to those genes (eg, granisetron could be used instead of ondansetron for acute nausea in a patient with a CYP2D6 ultrarapid metabolizer phenotype). This illustrates the potential longitudinal utility of pharmacogenetic test results from childhood even into adulthood, which can be further facilitated by enhanced EHR interoperability, enabling dissemination of laboratory results across health care systems.^37^ These opportunities for genotype-guided prescribing will likely go unrecognized unless pediatricians receive adequate education and training in pharmacogenetics.

Changes in prescriptions over time were most prominent for analgesics. Codeine use declined after the 2013 FDA public warning against using the drug after tonsillectomy and/or adenoidectomy^38^; the FDA added a contraindication to codeine use in children younger than 12 years in 2017.^39^ Some clinicians support *CYP2D6* testing to preserve codeine as an option for children by excluding those who have poor or ultrarapid metabolizer phenotypes and are therefore at increased risk for poor analgesic response or respiratory depression, respectively.^25,40^ The decrease in codeine prescriptions mirrors an increase in oxycodone use, approved for children 11 years or older in 2017^41^; its off-label use in children was common before that date and was generally considered acceptable. Compared with morphine, oxycodone is associated with a reduced frequency of adverse effects, particularly delirium.^42,43,44^ These characteristics, combined with increased liability concerns and lack of pharmacogenetic testing, suggest a shift in analgesic utilization toward oxycodone as a preferable option in pediatric patients in the US but not in Canada.^21^ Unlike with codeine, the *CYP2D6* genotype is not strongly associated with observed oxycodone response,^45,46,47^ but oxycodone and its active metabolite oxymorphone can lead to opioid dependence,^48,49^ raising reasonable concerns.

### Limitations

Several limitations of this study should be noted. Only CPIC level A medications and prespecified alternatives were extracted from the EHR; therefore, we have an incomplete picture of the prescribing patterns of other medications (eg, sertraline, which is included in a CPIC guideline^33^ but is level B). The CPIC level A designations change over time; since the initiation of this project, several additional medications commonly used in pediatric patients (eg, atomoxetine,^12^ ibuprofen,^50^ and proton pump inhibitors^51^) are now designated level A and have CPIC guidelines. On the other hand, oxycodone was designated as level A but has been downgraded owing to evolving evidence. Metabolizer status assignments are also evolving. Reclassification of individuals with CYP2D6 activity scores of 1 from normal to intermediate metabolizers, per recently updated *CYP2D6* translation guidelines,^18^ would result in a higher prevalence of actionable exposures to the associated medications. The generalizability of our findings is affected by the preponderance of academic medical centers; we used equal weighting across sites to avoid underrepresentation of the smaller, community-based health centers (relative to some of the very large academic medical centers). Individual site data may be more informative for some applications, given the variability across sites, and our efforts may not have led to a study population representative of all communities. Use of 1000 Genomes or other publicly available data^52^ to estimate actionability may also affect generalizability. Drug indications were not considered in our analysis, which may affect actionability and feasibility of pharmacogenomic implementation. Not all CPIC level A drugs have robust pediatric data, and extrapolation from adults to children warrants caution. Although activity for certain drug-metabolizing enzymes (eg, CYP2D6) is fully mature by early childhood, evidence suggests that activity for other enzymes (eg, CYP2C19) may be increased in children relative to adults.^53,54^ With respect to reuse of pharmacogenetic results over time, it would be of great interest to observe individual patients for longer than 1 year; however, our aggregated data precluded this analysis. The analyses included missing data from sites and some very low exposure prevalences requiring extrapolation, which may introduce error. Encounter data (denominator) and prescription data (numerator) required separate data extractions, precluding explicit confirmation that every individual with a prescription is represented in the encounter data.

## Conclusions

The findings of this serial cross-sectional study suggest that opportunities for pharmacogenetic implementation among pediatric patients in the US are abundant. For pediatric institutions interested in implementing pharmacogenetic testing, the utility will likely be greatest for *CYP2C19* and *CYP2D6*, particularly for the antiemetic ondansetron, analgesics, and antidepressants.

## Notes

Supplement. **eMethods.** Statistical Analysis Details **eTable 1.** CPIC Level A Drugs and Associated Genes Included in Study **eTable 2.** Characteristics of the Patient Populations and Sites’ Description **eFigure 1.** Annual Prevalence of Exposure to at Least 1 CPIC Level A Drug by Age Differs by Hospital Type **eFigure 2.** Prevalence of Exposure for Ondansetron and Escitalopram by Age **eFigure 3.** Prevalence of Exposure to Analgesics by Age Stratified by Hospital Type
