# Supplementary material for: Prescribing Prevalence of Medications With Potential Genotype-Guided Dosing in Pediatric Patients
Source: JAMA Netw Open. 2020 Dec 14;3(12):e2029411. doi: 10.1001/jamanetworkopen.2020.29411 (PMC7737091; doi:10.1001/jamanetworkopen.2020.29411)
Supplement: Supplement. — eMethods. Statistical Analysis Details eTable 1. CPIC Level A Drugs and Associated Genes Included in Study eTable 2. Characteristics of the Patient Populations and Sites’ Description eFigure 1. Annual Prevalence of Exposure to at Least 1 CPIC Level A Drug by Age Differs by Hospital Type eFigure 2. Prevalence of Exposure for Ondansetron and Escitalopram by Age eFigure 3. Prevalence of Exposure to Analgesics by Age Stratified by Hospital Type [file jamanetwopen-e2029411-s001.pdf]

# Supplementary Online Content

Ramsey LB, Ong HH, Schildcrout JS, et al; IGNITE Pharmacogenetics Working Group. Prescribing prevalence of medications with potential genotype-guided dosing in pediatric patients. *JAMA Netw Open*. 2020;3(12):e2029411. doi:10.1001/jamanetworkopen.2020.29411

**eMethods.** Statistical Analysis Details

**eTable 1.** CPIC Level A Drugs and Associated Genes Included in Study

**eTable 2.** Characteristics of the Patient Populations and Sites' Description

**eFigure 1.** Annual Prevalence of Exposure to at Least 1 CPIC Level A Drug by Age Differs by Hospital Type

**eFigure 2.** Prevalence of Exposure for Ondansetron and Escitalopram by Age

**eFigure 3.** Prevalence of Exposure to Analgesics by Age Stratified by Hospital Type

This supplementary material has been provided by the authors to give readers additional information about their work.

## eMethods. Statistical Analysis Details

*Prescribing patterns over time*

We examine prescribing prevalences from 2011 to 2017, separately, for a) any CPIC level A medication, b) at least one, two, three and four CPIC level A medications, c) distinct classes of CPIC level A medications (e.g., analgesic, statins, anticoagulants), d) for individual CPIC level A medications, and e) for medications combined with associated metabolizing genes. For each model, we conducted the logistic regression analysis for binomial data of the form,

$$\log\left(\frac{p_s(t)}{1-p_s(t)}\right) \equiv lo_s(t) = \alpha_0 + f_1(t)\alpha_t + I(Site = s)\alpha_s + f_2(t)I(Site = s)\alpha_{st}.$$

where $p_s(t)$ is the prescription prevalence at site s during year t, $lo_s(t)$ is the corresponding log-odds, and $I(Site = s)$ is an indicator variable that is set to 1 if $Site = s$ and is 0 otherwise. With 16 sites, we included 15 indicator variables. Functions $f_1(t)$ and $f_2(t)$ are flexible spline functions of the year variable (t=2011, 2012, … 2017) that together allow the log-odds to change over time in a non-linear fashion at each site separately. From this model, we were able to estimate the site-specific medication prescription prevalences for each year which we combined across sites to obtain overall, annual prescription prevalences. Because there was dramatic site-to-site variability in sample sizes (see Table 1) we considered two distinct weighting procedures to combine site-year prevalences across sites to estimate overall prevalences each year (t): 1) by site weighting and 2) by patient weighting, the latter as a sensitivity analysis. With by site weighting, we combined site-year estimates across sites by using a simple average. Thus, for S=16 total sites, the equal site weighted average log-odds is given by,

$$\widehat{lo}^{SW}(t) = \frac{1}{S}\sum_{s=1}^{S}\widehat{lo}_s(t)$$

©2020 Ramsey L et al. *JAMA Network Open*.

and the estimated variance is given by,

$$\widehat{Var}[\widehat{lo}^{SW}(t)] = \frac{1}{S^2}\sum_{s=1}^{S}\widehat{Var}[\widehat{lo}_s(t)].$$

The log-odds and associated confidence intervals are converted to risks using the inverse of the logistic function. By giving each site equal weight, we assume the perspective that there exists a population of sites from which 16 were sampled and contributed data to this analysis. The risks reported in the main text are based on by site weights. With by patient weighting, we combined site-year estimates using weights that are proportional to sites' sample sizes. With $N_s(t)$ patients at site $s$ in year $t$ and $N_{tot}(t) = \sum_{s=1}^{S} N_s(t)$ total patients observed in year $t$, the by patient weighted estimated log-odds of medication prescription at year $t$ is given by,

$$\widehat{lo}^{PW}(t) = \sum_{s=1}^{S}\frac{N_s(t)}{N_{tot}(t)}\widehat{lo}_s(t)$$

With an estimated variance,

$$\widehat{Var}[\widehat{lo}^{PW}(t)] = \sum_{s=1}^{S}\left(\frac{N_s(t)}{N_{tot}(t)}\right)^2\widehat{Var}\left[\widehat{lo}_s(t)\right].$$

Intuitively, by patient weighting gives equal weight to each patient in the analysis, and so in contrast to by site weighting, larger sites are more heavily weighted than smaller sites when calculating annual prevalences across sites.

To capture the overall prescribing prevalences during each year, we sought to include all sites in the calculation of the by site weighted and by patient weighted averages; however, several sites were missing annual prescribing data (see Table S2). Because the availability, or lack thereof, of prescribing data relied upon an operational and compatible electronic health record system, and was unlikely to be due to prescribing patterns themselves, we assumed the

data were missing at random when sites were missing data for one or two consecutive years. To avoid excessive extrapolation of observed time trends, for sites with missing prescribing data in more than two consecutive years, we removed the time trend for the site and estimated a single, across-year prevalence. Finally, site-specific estimates were unstable when prevalences were extremely low (e.g., for medications with very low prescribing prevalences). If there were less than 20 prescriptions for a medication in any year, we also removed the time trend and estimated a single, across-year prevalence.

*Prescribing patterns by demographic characteristics*

To examine the prescribing patterns by gender, race, and age, we fit and summarized logistic regression models similar to those described above. To estimate prescribing patterns across the age distribution, we substituted age in for the year variable using restricted cubic spline functions to permit non-linear age trends. To capture prescribing patterns for each of gender and race we combined site-specific estimates from the model

$$\log\left(\frac{p_s(d)}{1-p_s(d)}\right) \equiv lo_d(t) = \alpha_0 + I(D=d)\alpha_d + I(Site=s)\alpha_s + I(D=d)I(Site=s)\alpha_{sd}$$

where $I(D=d)$ is an indicator variable for a demographic subgroup $d$ (e.g., male, female, white, black, etc.).

## eTable 1. CPIC Level A Drugs and Associated Genes Included in Study

| Medication Name | Drug Class** | Gene(s) | GenBank ID(s) | Alternative Drugs |
|---|---|---|---|---|
| Abacavir | HIV antivirals | *HLA-B*57:01* | 3106 | |
| Allopurinol | other | *HLA-B*58:01* | 3106 | |
| Amitriptyline | TCA | *CYP2C19, CYP2D6* | 1557, 1565 | |
| Atazanavir | HIV antivirals | *UGT1A1* | 54685 | |
| Azathioprine | immunosuppressant | *TPMT, NUDT15* | 7172, 55270 | |
| Capecitabine | chemotherapeutic | *DPYD* | 1806 | |
| Carbamazepine | anti-seizure | *HLA-B*15:02, HLA-A*31:01* | 3106, 3105 | |
| Citalopram | SSRI | *CYP2C19* | 1557 | |
| Clopidogrel | -- | *CYP2C19* | 1557 | prasugrel, ticagrelor |
| Codeine | analgesic | *CYP2D6* | 1565 | |
| Desflurane | anesthetic | *RYR1, CACNA1S* | 6261, 779 | |
| Escitalopram | SSRI | *CYP2C19* | 1557 | |
| Fluorouracil | chemotherapeutic | *DPYD* | 1806 | |
| Fluvoxamine | SSRI | *CYP2D6* | 1565 | |
| Irinotecan | chemotherapeutic | *UGT1A1* | 54685 | |
| Isoflurane | anesthetic | *RYR1, CACNA1S* | 6261, 779 | |
| Ivacaftor | other | *CFTR* | 1080 | |
| Mercaptopurine | immunosuppressant | *TPMT, NUDT15* | 7172, 55270 | |
| Nortriptyline | TCA | *CYP2D6* | 1565 | |
| Ondansetron* | -- | *CYP2D6* | 1565 | |
| Oxcarbazepine | anti-seizure | *HLA-B*15:02* | 3106 | |
| Oxycodone | analgesic | *CYP2D6* | 1565 | |
| Paroxetine | SSRI | *CYP2D6* | 1565 | |
| Peginterferon | hepatitis C antivirals | *IFNL3* | 282617 | |
| Peginterferon Alfa-2a | hepatitis C antivirals | *IFNL3* | 282617 | |
| Peginterferon Alfa-2b | hepatitis C antivirals | *IFNL3* | 282617 | |
| Phenytoin | anti-seizure | *HLA-B*15:02, CYP2C9* | 3106, 1559 | |
| Rasburicase | other | *G6PD* | 2539 | |
| Ribavirin | hepatitis C antivirals | *IFNL3* | 282617 | |
| Sevoflurane | anesthetic | *RYR1, CACNA1S* | 6261, 779 | |
| Simvastatin | -- | *SLCO1B1* | 10599 | atorvastatin, fluvastatin, lovastatin, pitavastatin, pravastatin, rosuvastatin |
| Succinylcholine | anesthetic | *RYR1, CACNA1S* | 6261, 779 | |
| Tacrolimus | immunosuppressant | *CYP3A5* | 1577 | |
| Tamoxifen | -- | *CYP2D6* | 1565 | |
| Thioguanine | immunosuppressant | *TPMT, NUDT15* | 7172, 55270 | |
| Tramadol | analgesic | *CYP2D6* | 1565 | |
| Voriconazole | -- | *CYP2C19* | 1557 | |
| Warfarin | -- | *CYP2C9, VKORC1* | 1559, 79001 | dabigatran, apixaban, rivaroxaban |
| *The antiemetic tropisetron is also a CPIC level A drug, but not available in the US. **No drug class is listed when there is only one CPIC level A drug in the class. | | | | |

©2020 Ramsey L et al. *JAMA Network Open*.

## eTable 2. Characteristics of the Patient Populations and Sites' Description

**Demographics**

| Site | 1 | 2 | 3 | 4 | 5 | 6 | 7 | 8 | 9 | 10 | 11 | 12 | 13 | 14 | 15 | 16 |
|---|---|---|---|---|---|---|---|---|---|---|---|---|---|---|---|---|
| Age in years (median, IQR) | 9.0 4.0-15.5 | 7.0 3.0-12.0 | 8.0 4.0-14.0 | 7.0 3.0-13.0 | 8.0 3.0-14.0 | 10.0 4.0-16.0 | 9.0 4.0-14.0 | 19.0 17.0-20.0 | 18.0 16.0-19.0 | 16.0 8.0-19.0 | 15.5 10.0-18.0 | 6.0 3.0-12.0 | 10.0 5.0-14.5 | 18.8 17.0-20.0 | 15.0 9.0-18.0 | 13.0 4.5-18.0 |
| Female (%) | 48.5 | 49.9 | 48.3 | 49.7 | 50.7 | 50.7 | 52.3 | 67.7 | 62.4 | 60.7 | 58.3 | 47.4 | 48.2 | 66.7 | 58.1 | 49.8 |
| White (%) | 41.5 | 82.9 | 74.0 | 74.7 | 64.7 | 72.8 | 22.0 | 53.9 | 12.2 | 23.2 | 86.9 | 62.8 | 61.9 | 50.0 | 74.1 | 51.7 |
| African American (%) | 7.4 | 8.2 | 15.0 | 18.7 | 7.4 | 18.5 | 17.9 | 33.9 | 70.2 | 34.9 | 9.4 | 6.8 | 18.1 | 42.3 | 11.9 | 33.7 |
| Asian (%) | 11.3 | 0.4 | 1.4 | 1.6 | 1.6 | 2.3 | 1.2 | 1.1 | 0.8 | 0.2 | 0.5 | 2.5 | 1.4 | 0.9 | 4.1 | 2.4 |
| American Indian or Native Alaskan (%) | 0.7 | 0.1 | 0.1 | 0.2 | 0.6 | 0.4 | 0.3 | 0.2 | 0.0 | 0.2 | 0.2 | 0.6 | 0.2 | 0.1 | 1.0 | 0.7 |
| Pacific Islander | 1.1 | 0.0 | 0.1 | 0.1 | 0.4 | 0.0 | 0.0 | 0.1 | 0.0 | 0.2 | 0.1 | 0.2 | 0.1 | 0.0 | 0.2 | 0.0 |
| Unknown/ Other (%) | 38.2 | 8.4 | 8.9 | 4.6 | 25.8 | 5.8 | 58.2 | 10.1 | 17.3 | 40.8 | 2.6 | 27.3 | 17.1 | 7.3 | 9.4 | 12.1 |

**Site characteristics**

| Site | 1 | 2 | 3 | 4 | 5 | 6 | 7 | 8 | 9 | 10 | 11 | 12 | 13 | 14 | 15 | 16 |
|---|---|---|---|---|---|---|---|---|---|---|---|---|---|---|---|---|
| Annual No. patients with encounters (median)[a] | 18,726 | 85,222 | 165,658 | 221,172 | 102,945 | 51,914 | 77,893 | 69,682 | 4,790 | 44,032 | 799,964 | 234,305 | 376,515 | 90,249 | 172,802 | 351,018 |
| Annual No. patients with target Rx (median)[b] | 6,216 | 24,187 | 37,053 | 38,230 | 13,364 | 5,160 | 2,984 | 2,870 | 238 | 2,382 | 10,803 | 29,380 | 11,985 | 2,843 | 5,897 | 3,817 |
| Years included in data | 2012-2017 | 2013-2017 | 2012-2017 | 2011-2015 | 2011-2017 | 2011-2016 | 2011-2017 | 2012-2016 | 2016-2017 | 2011, 2014-2016 | 2011-2015 | 2011-2017 | 2011-2017 | 2011-2017 | 2011-2017 | 2011-2015 |
| Clinical setting | AMC | CH | AMC | AMC | AMC | AMC | AMC | AMC | CH | CH | AMC | AMC | CH | AMC/ CH | AMC/ CH | AMC |
| Organization | Health System | Health System | Health System | Individual Hospital | Health System | Health System | Health System | Health System | Individual Hospital | Health System | Health System | Individual Hospital | Health System | Health System | Health System | Health System |
| Facility Structure | FSPH | FSPH | PS | FSPH | PS | PS, FSPH | PS | PS | PS | PS | PS | FSPH | FSPH | PS | PS | PS |
| No. licensed beds | 373 | 309 | 687 | 315 | 1,620 | 267 | 3,815 | 2,940 | 114 | Up to 350 | 8,400 | 479 | 390 | 1,157 | 2,071 | 2,578 |

©2020 Ramsey L et al. *JAMA Network Open*.

| | | | | | | | | | | | | | | | | |
|---|---|---|---|---|---|---|---|---|---|---|---|---|---|---|---|---|
| No. ambulatory visits | 1,149,883 | 269,468 | 1,050,970 | > 1,000,000 | 2,574,489 | 334,956 | > 3,499,000 | 5,747,434 | 40,621 | 952,851 | 4,700,000 | 574,929 | 823,660 | 1,300,000 | > 2,100,000 | 1,530,470 |
| No. Emergency room visits | 98,526 | 89,935 | 179,857 | 176,926 | 356,946 | 49,141 | 536,443 | 359,568 | 26,410 | 102,950 | 957,000 | 173,085 | 79,508 | 103,743 | 326,198 | 453,362 |
| Surgical procedures | 38,306 | 3,042 | 35,292 | 24,135 | 66,111 | 17,678 | N/A | 77,869 | N/A | 10,764 | 220,000 | 19,362 | 18,158 | 20,000 | 90,411 | 75,274 |
| EHR vendor | Epic | Cerner | Epic | Cerner | Epic | Other | Epic | Epic/ Other | Other | Epic/ Other | Epic | Epic | Epic | Cerner | Epic | Epic |
| Changes in EHR | No | No | No | No | No | No | No | No | No | Yes | No | No | No | No | No | Yes |
| **Encounter and medication data pull** | | | | | | | | | | | | | | | | |
| Site | 1 | 2 | 3 | 4 | 5 | 6 | 7 | 8 | 9 | 10 | 11 | 12 | 13 | 14 | 15 | 16 |
| Encounter type | Inpt, Outpt, ED | Inpt, Outpt, ED | Inpt, Outpt, ED | Outpt, ED | Inpt, Outpt, ED | Inpt, Outpt | Inpt, Outpt, ED | Inpt, Outpt, ED | Inpt, Outpt, ED | Inpt, Outpt, ED | Outpt, ED | Inpt, Outpt, ED | Inpt, Outpt, ED | Inpt, Outpt, ED | Inpt, Outpt, ED | Inpt, Outpt, ED |
| Encounter data pull | Manual review | Manual review | Manual review | Encoun-ter filtering | Manual review | Encoun-ter filtering | Manual review | Manual review | Manual review | Manual review | Encoun-ter filtering | Manual review | Manual review | Manual review | Manual review | Manual review |
| Medication query | Epic clarity ID | Med expan-sion | Trade, generic names/ RxNorm ID | Med expan-sion | Med expan-sion | Med expan-sion | Trade, generic names | Med expan-sion | Med expan-sion | RxNorm ID | Med expan-sion | Med expan-sion | Epic med Clarity ID, trade, generic names | Med expan-sion | Med expan-sion | RxNorm ID |
| No. of Target Medications | 40 | 34 | 44 | 40 | 39 | 39 | 44 | 42 | 11 | 34 | 36 | 43 | 42 | 42 | 41 | 31 |

Abbreviations: No. : Number; a and b: denotes unique patients; Rx: Prescriptions; IQR: Interquartile range; AMC: Academic Medical Center, CH: Community Hospital; PS: Pediatric service within adult care Health System, FSPH: Free Standing Pediatric Hospital; EHR: Electronic Health Records; Changes in EHR denotes any changes in EHR during study years for that site; Inpt: Inpatient; Outpt: Outpatient; ED: Emergency Department; Encounter filtering: Encounter filter based on prescription rate (sites 4 and 11) or inpatient, outpatient class type (site 6); Med: Medication; Med expansion: Querying and extracting medication data using an extended generic name list; Target Medication: CPIC Level A or alternative medications; N/A: not available.

©2020 Ramsey L et al. *JAMA Network Open*.

**eFigure 1. Annual Prevalence of Exposure to at Least 1 CPIC Level A Drug by Age Differs by Hospital Type**

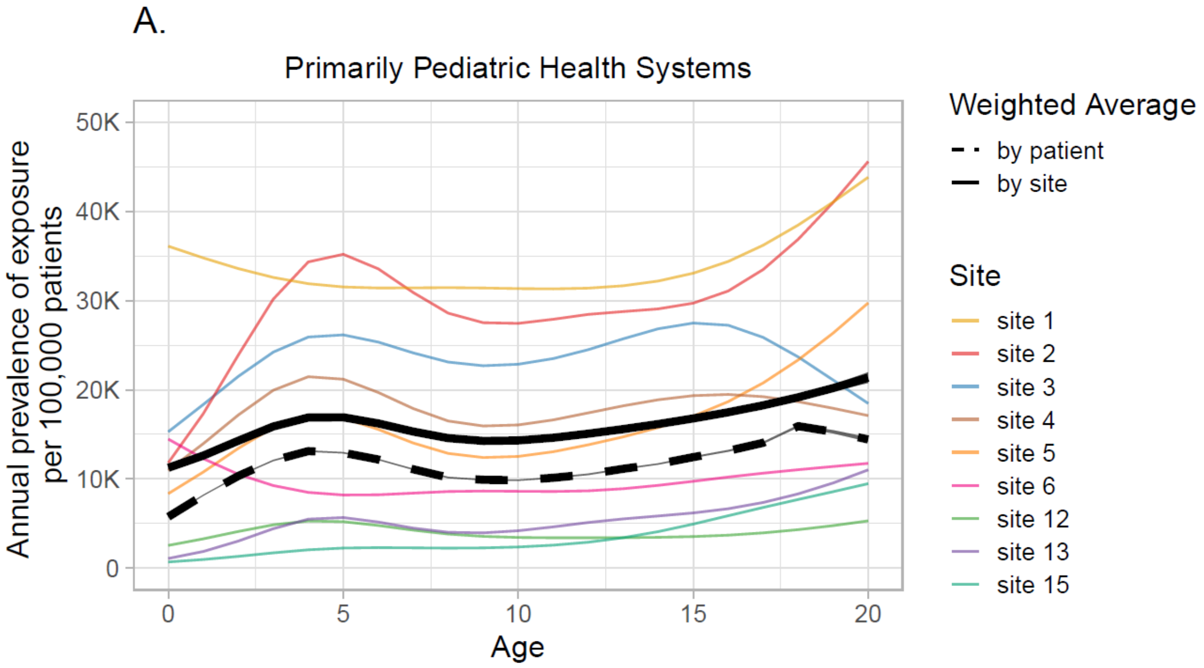


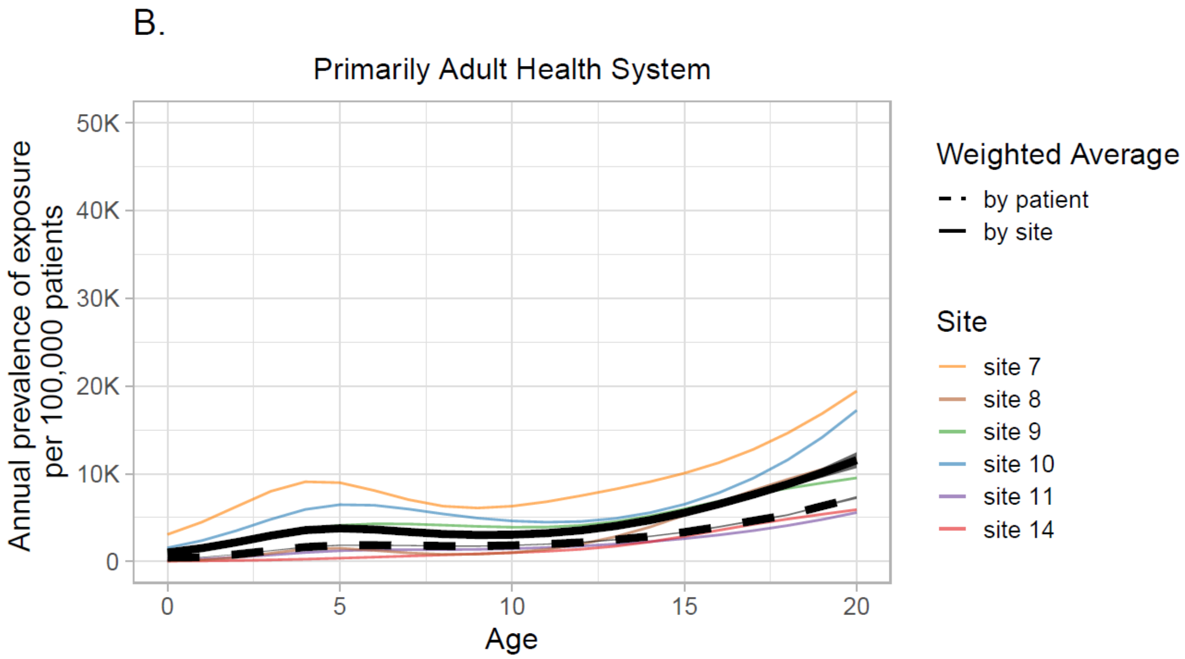


Sites identified themselves as primarily pediatric (A) or adult health systems (B). One site did not have demographic data available for encounters and is excluded from these analyses. The annual prevalence of exposure is shown for 2015. The mean prevalence of exposure across all sites was calculated in two ways: equal site weighting (solid black line) and proportional to site size weighting (dashed black line). The 95% confidence bands for the two means are filled in gray but may be too narrow to be observed.

©2020 Ramsey L et al. *JAMA Network Open*.

eFigure 2. Prevalence of Exposure for Ondansetron and Escitalopram by Age

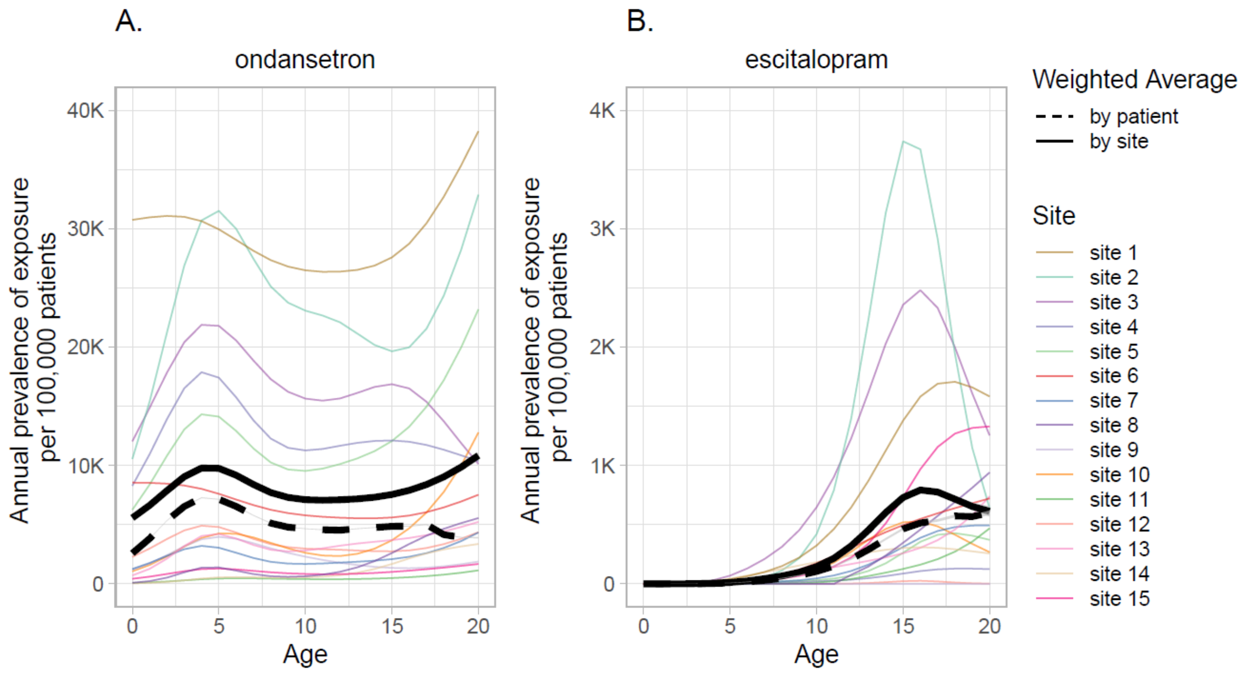


The mean prevalence of exposure across all sites was calculated in two ways: equal site weighting (solid black line) and proportional to site size weighting (dashed black line). One site did not have demographic data available for encounters and is excluded from these analyses. The 95% confidence bands for the two means are filled in gray but may be too narrow to be observed.

©2020 Ramsey L et al. *JAMA Network Open*.

## eFigure 3. Prevalence of Exposure to Analgesics by Age Stratified by Hospital Type

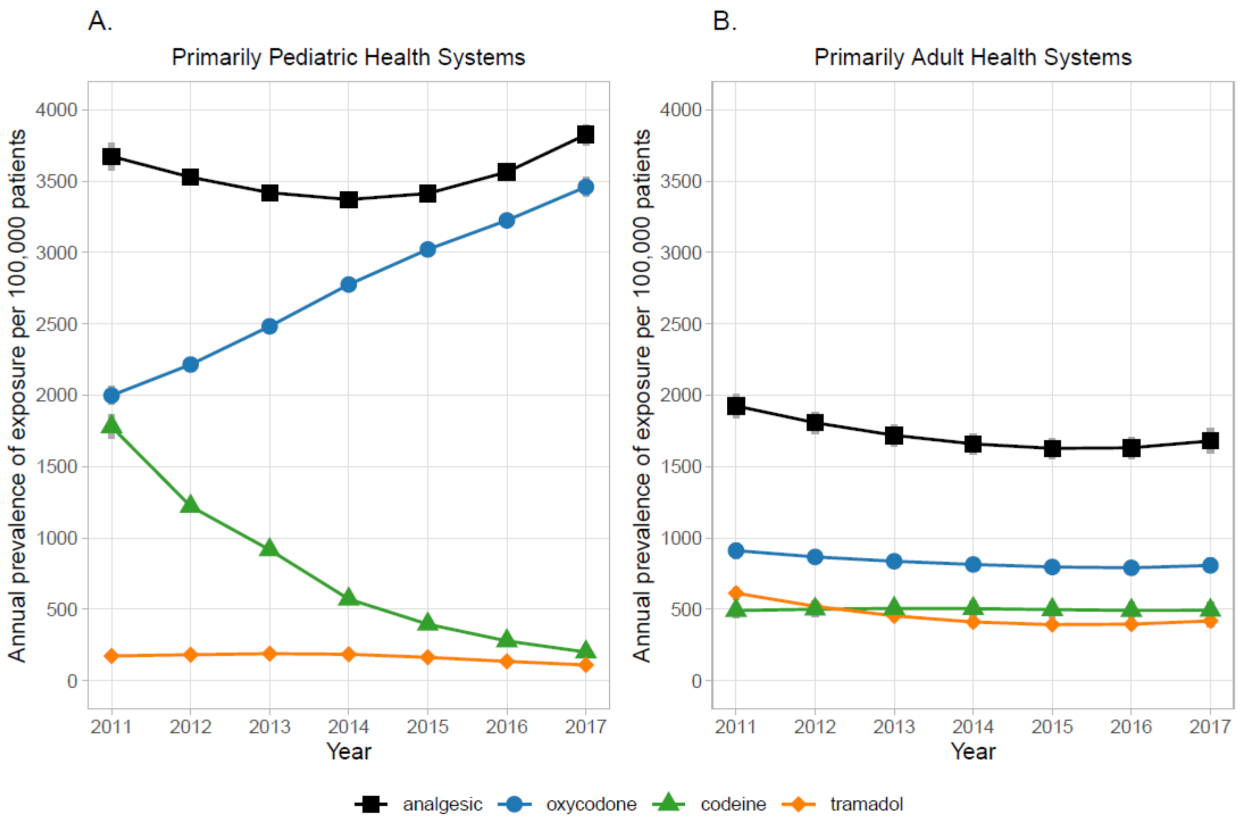


Annual prevalence of exposure for analgesics was averaged using equal site weighting. A, only primarily pediatric health systems. B, only primarily adult health systems. The estimated prevalence of exposure for the analgesic (black line) is taken from the drug class model, whereas those for oxycodone, codeine, and tramadol are taken from the individual drug model. The 95% confidence bands for the means are filled in gray but may be too narrow to be observed.

©2020 Ramsey L et al. *JAMA Network Open*.
